# Interval Split Covariance Intersection Filter: Theory and Its Application to Cooperative Localization in a Multi-Sensor Multi-Vehicle System

**DOI:** 10.3390/s24103124

**Published:** 2024-05-14

**Authors:** Xiaoyu Shan, Adnane Cabani, Houcine Chafouk

**Affiliations:** ESIGELEC, IRSEEM, Université de Rouen Normandie, 76000 Rouen, France; xiaoyu.shan@esigelec.fr

**Keywords:** data incest, multi-sensor multi-vehicle (MSMV) system, cooperative localization, interval analysis, split covariance intersection filter (SCIF)

## Abstract

The data incest problem causes inter-estimate correlation during data fusion processes, which yields inconsistent data fusion results. Especially in the multi-sensor multi-vehicle (MSMV) system, the data incest problem is serious due to multiple relative position estimations, which not only lead to pessimistic estimation but also cause additional computational overhead. In order to address the data incest problem, we propose a new data fusion method termed the interval split covariance intersection filter (ISCIF). The general consistency of the ISCIF is proven, serving as supplementary proof for the split covariance intersection filter (SCIF). Moreover, a decentralized MSMV localization system including absolute and relative positioning stages is designed. In the absolute positioning stage, each vehicle uses the ISCIF algorithm to update its own position based on absolute measurements. In the relative position stage, the interval constraint propagation (ICP) method is implemented to preprocess multiple relative position estimates and initially prepare input data for ISCIF. Then, the proposed ISCIF algorithm is employed to realize relative positioning. In addition, comparative simulations demonstrate that the proposed method can achieve both accurate and consistent results compared with the state-of-the-art methods.

## 1. Introduction

Localization plays an important role in autonomous driving and intelligent transportation systems (ITSs). Traditional sensor-based vehicle localization methods (including GPS-based methods [1], inertial measurement unit (IMU)-based methods [2], camera-based methods [3], etc.) can reach high-performance localization in certain scenarios. However, traditional vehicle localization methods have obvious drawbacks. For example, the accuracy of GPS-based methods can be affected easily by the environment, the cumulative errors can seriously affect the accuracy of IMU-based methods during long-distance localization processes, and camera-based methods normally need additional overhead for map and data storage. With the rapid development of the vehicle-to-vehicle (V2V) and vehicle-to-infrastructure (V2I) communication techniques, data sharing is enabled among vehicles and infrastructure in the system. By using V2V [4] and V2I [5,6] communication techniques, cooperative vehicle localization can benefit from data sharing, which has better performance than traditional localization methods. Many data fusion methods like the unscented Kalman filter (UKF) [7], the extended Kalman filter (EKF) [8], and the particle filter (PF) [9] have already been proposed to realize cooperative vehicle localization. More details about vehicle localization have been highlighted in [10,11].

**Figure 1:**
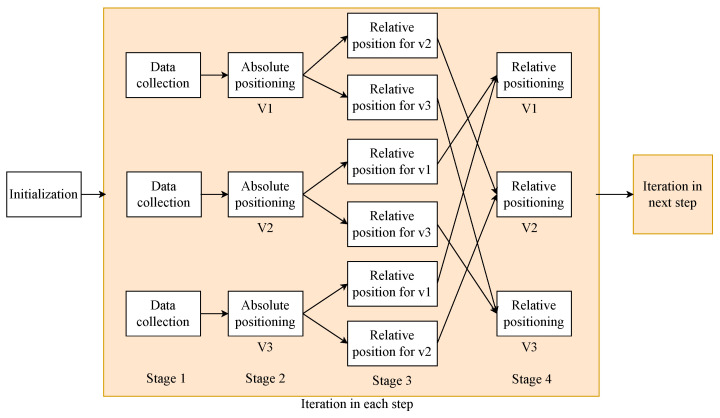
The process of cooperative vehicle localization in an MSMV system.

In the cooperative localization domain, one significant challenge is to solve the data incest problem. It is caused by the multiple usage of identical information, and these items of information are considered independent from each other [12]. Furthermore, inter-estimate correlation among different estimate sources is caused by data incest [13], which yields inconsistent estimates and an over-convergence problem [14]. The data incest problem arises particularly in scenarios where the relative positions among vehicles are used. Specifically, when there are multiple relative measurements, the data incest problem becomes more serious due to the complex data flow [15]. The process of the cooperative vehicle localization in the MSMV system is shown in Figure 1. After the initialization, in each iteration step, there are four stages for each vehicle: data collection, absolute positioning, providing relative measurements for their neighbors by data sharing, and relative positioning when each vehicle receives its relative measurements. When three vehicles communicate and share relative measurements with each other, each vehicle in the system may face the data incest problem in the relative positioning stage from the second iteration process because the relative estimates calculated by relative measurements provided by neighboring vehicles may have correlation with their own state. The data incest problem causes inconsistent results during the data fusion process due to the inter-estimate correlation. In order to address the data incest problem, one method called the covariance intersection (CI) filter has been proposed in [16]. However, the CI algorithm does not distinguish between the independent and correlated parts in the estimated resources, which will negatively affect the accuracy of the fusion result.

In order to further increase the accuracy, another approach termed the split covariance intersection filter (SCIF) has been reported in [14]. The independent and correlated parts of fusion resources are both considered, which can yield consistent estimates. However, in the multi-sensor multi-vehicle (MSMV) system, there are multiple relative measurements when one vehicle is observed by different neighbors. The traditional SCIF is not suitable because the state vector is updated frequently by using multiple relative position data points, and the quality of the relative position information cannot be guaranteed, which may yield pessimistic estimates and unnecessary time overhead.

Furthermore, the interval constraints propagation (ICP) is an effective fusion method that has been widely used in the vehicle and robot localization domain [17]. It uses communication techniques to propagate the possible constraints and achieve data fusion based on these restrictions, and it has good localization performance especially when faced with redundant data. Moreover, it can avoid the over-convergence problem by analyzing the values within the confidence domain. However, it is difficult to decide the weight of each constraint in the real localization application. In other words, when different constraints are considered to have the same weight, the results will have the problem of loss of accuracy. In addition, the interval Kalman filter (IKF) is proposed to increase the accuracy [18]. However, the inversion of the interval covariance matrix can only be solved approximately, and it is an NP-hard problem [19].

Additionally, the two-layer data fusion method has been proposed to solve the data incest problem [20]. However, the centralized system architecture with only one fusion center yields high computational overhead, and this problem will be more serious in future large-scale vehicle systems [14]. In order to address this kind of problem, a decentralized architecture is employed in our designed system in order to reduce the computational burden on the system.

Our contributions in this paper are as follows:

- Motivated by addressing the problem caused by the inter-estimate correlation (data incest) and avoiding problems caused by inverse of interval matrix in IKF, a new data fusion method called ISCIF is proposed.
- ISCIF incorporating the ICP mechanism for cooperative vehicle localization in the MSMV system is presented. The ICP method is employed to preprocess multiple relative estimates and provide input data for ISCIF.
- Different from contributions in our previous work [15], the consistency of the proposed ISCIF is given. Additionally, a more complex simulation scenario is designed to illustrate the accuracy of the proposed cooperative vehicle localization method.

This paper is organized as follows. Related techniques and the ISCIF method are proposed in Section 2. MSMV cooperative vehicle localization is proposed in Section 3. Simulation-based experimental results are detailed in Section 4, and the conclusion and future work follow in Section 5.

## 2. Related Techniques and Proposed ISCIF

Data fusion methods based on interval analysis, such as interval constraint propagation (ICP) and interval Kalman filter (IKF), are effective techniques for interval-based vehicle localization systems. We briefly present these methods and propose the interval split covariance intersection filter (ISCIF) in this section.

### 2.1. Interval Analysis

Interval analysis is a numerical method that can provide guaranteed values by representing numbers as intervals. An interval is represented using the notation [a], which includes the lower bound $\underline{a}$ and the upper bound $\bar{a}$:

(1) $$\left[a\right]=\left[\underline{a},\bar{a}\right]=\left\{a\epsilon R,\underline{a}\leq a\leq \bar{a}\right\}.$$

The width of an interval can be calculated by Equation (2), and the midpoint of an interval is denoted in Equation (3):

(2) $$width\left(\left[a\right]\right)=\bar{a}-\underline{a},$$

(3) $$mid\left(\left[a\right]\right)=width\left(\left[a\right]\right)/2+\underline{a}.$$

An interval vector, a box, is the Cartesian product of *n* intervals, which can be denoted as:

(4) $$\left[A\right]=\left[a_{1}\right]\times \left[a_{2}\right]\times \cdots \times \left[a_{n}\right].$$

Assuming there are two intervals [a] and [b], the addition and subtraction operations can be denoted as follows:

(5) $$\left[a\right]+\left[b\right]=\left[\underline{a}+\underline{b},\bar{a}+\bar{b}\right],$$

(6) $$\left[a\right]-\left[b\right]=\left[\underline{a}-\bar{b},\bar{a}-\underline{b}\right].$$

**Figure 2:**
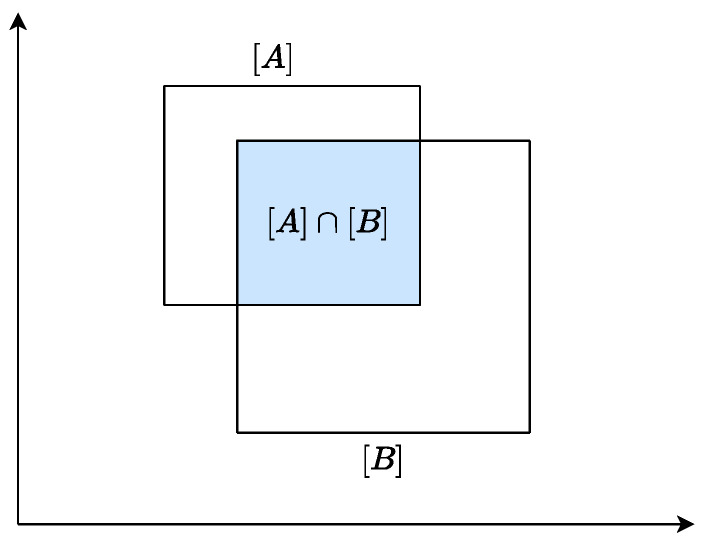
Intersection between two boxes.

Moreover, the intersection operation is defined in Equation (7). The intersection operation of two boxes is shown in Figure 2. Each component in the two boxes is involved in the operation according to the defined rules.

(7) $$\left\{c\in \left[A\right]\&\&\;c\in \left[B\right]\right\}=\left[A\right]\cap \left[B\right]$$

In addition, the inclusion function of $f:R^{a}\to R^{b}$ has been defined as $\left[f\right]$ to realize arithmetical operations and elementary functions in the function f(). Note that the inclusion function is also suitable for the box $\left[A\right]$.

(8) $$f\left(\left[A\right]\right)\in \left[f\right]\left(\left[A\right]\right),\forall \left[A\right]\in R^{a}$$

In order to create an inclusion function, one popular method is to replace all the variables and operators in the original functions with their interval values [21]. For example, the inclusion function of the function $f\left(x\right)=x^{3}+x^{2}-x$ is $\left[f\right]\left(\left[x\right]\right)={\left[x\right]}^{3}+{\left[x\right]}^{2}-\left[x\right]$. Note that the inclusion function can provide us equations denoted by interval variables (also called constraints), which play an important role in solving interval constraint satisfaction problems.

### 2.2. Interval Constraint Satisfaction Problem (ICSP) and Interval Constraint Propagation (ICP)

The result of one interval constraint satisfaction problem (ICSP) should satisfy all the constraints at the same time. One ICSP P, considering one box $\left[A\right]$, which includes *n* variables and *m* relations that link these variables, can be denoted as follows:

(9) $$P:f_{j}\left(a_{1},a_{2},\cdots ,a_{n}\right)=0,j=1,2,\cdots ,m.$$

**Figure 3:**
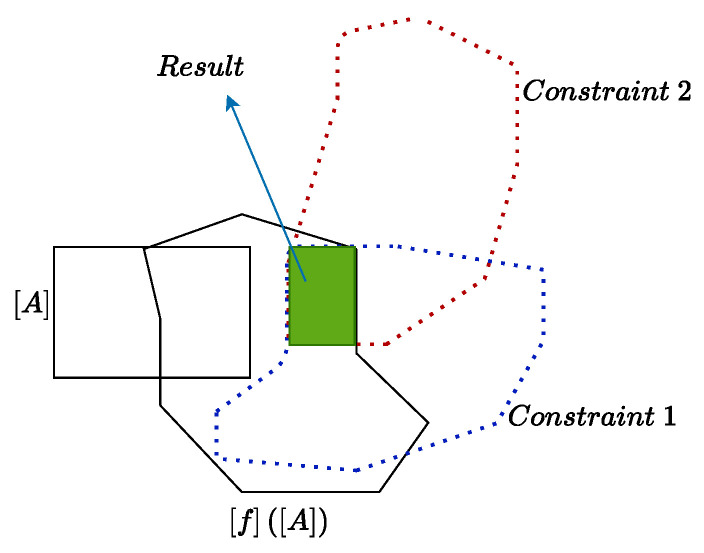
Contraction of one box by the contractor including two constraints.

Each variable is an interval, which can be represented by $\left[a_{i}\right],i=1,2\cdots n$. The interval constraint propagation (ICP) algorithm can solve the ICSP in a consistent way [22]. By developing a contractor, the domain of interval variables is reduced in the case where all constraints are satisfied, as shown in Figure 3.

Specifically, in MSMV localization system, the ICSP solved by the ICP method can be denoted as:

(10) $$P1:\left\{\left[X_{i}^{pre}\left(t\right)\right]\cap \left[X_{i}^{abs}\left(t\right)\right]\cap \left[X_{i}^{1}\right]\cap \cdots \cap \left[X_{i}^{m}\right]\right\},$$

where $\left[X_{i}^{pre}\left(t\right)\right]$ is the prediction box, $\left[X_{i}^{abs}\left(t\right)\right]$ is the absolute measurement box, and $\left[X_{i}^{1}\left(t\right)\right]\cdots ,\left[X_{i}^{m}\left(t\right)\right]$ are *m* relative estimate boxes that are calculated based on relative measurements provided by *m* neighbors when m≥2. Since the complexity of the ICP algorithm depends on the dimension of the box, for the purpose of reducing the computation burden, the ICP method is used to calculate the position on both *x* and *y* axes by processing multiple relative estimates for each vehicle in our paper, which reduces the box dimension in [22,23]. The result $\left[x_{i}^{R}\right]$ obtained by the ICP method for all relative estimates can be denoted as follows:

(11) $$\left[x_{i}^{R}\right]=\left\{\begin{array}{c} \left[x_{i}^{1}\left(t\right)\right]\\ \left[y_{i}^{1}\left(t\right)\right] \end{array}\right\}\cap \cdots \cap \left\{\begin{array}{c} \left[x_{i}^{m}\left(t\right)\right]\\ \left[y_{i}^{m}\left(t\right)\right] \end{array}\right\},$$

where the $x_{i}^{m}\left(t\right)$ and $y_{i}^{m}\left(t\right)$ are the relative position of vehicle *i* provided by vehicle *m* on the *x* and *y* axes, respectively.

### 2.3. Interval Split Covariance Intersection Filter (ISCIF)

In the MSMV cooperative localization system, when one vehicle utilizes relative measurements provided by neighboring vehicles for positioning, the estimated relative positions based on relative measurements may correlate with its own state. Consequently, the data to be fused are not independent of each other. Interval-based multi-sensor data fusion methods can effectively address the problem of unknown correlation between data to be fused [24]. One popular interval-based data fusion method is interval Kalman filter (IKF) [18] algorithm. It has the same statistical assumptions about noise and recursive structure as the traditional KF algorithm, and the process of IKF is presented as follows.

The interval discrete-time-controlled process is shown in Equation (12), where $\left[A\right]$, $\left[B\right]$, and $\left[H\right]$ are interval matrices, and the outputted results in each step are also intervals or boxes.

(12) $$\left\{\begin{array}{ccc} \left[x_{k}\right] & = & \left[A\right]\left[x_{k-1}\right]+\left[B\right]\left[U_{k-1}\right]+w_{k-1}\\ \left[Z_{k}\right] & = & \left[H\right]\left[x_{k}\right]+v_{k} \end{array}\right.$$

For the IKF method, the recursive structure is the same as that in the traditional KF, and the prediction and correction steps are represented by Equation (13) and Equation (14), respectively.

(13) $$\left\{\begin{array}{cc} \left[\hat{x_{k}^{-}}\right] & =\left[A\right]\left[\hat{x_{k-1}}\right]+\left[B\right]\left[U_{k-1}\right]\\ \left[P_{k}^{-}\right] & =\left[A\right]\left[P_{k-1}\right]{\left[A\right]}^{T}+Q \end{array}\right.$$

(14) $$\left\{\begin{array}{cc} \left[K_{k}\right] & =\left[P_{k}^{-}\right]{\left[H\right]}^{T}{\left(\left[H\right]\left[P_{k}^{-1}\right]{\left[H\right]}^{T}+R\right)}^{-1}\\ \hat{\left[x_{k}\right]} & =\hat{\left[x_{k}^{1}\right]}+\left[K_{k}\right]\left(\left[Z_{k}\right]-\left[H\right]\hat{\left[x_{k}^{-}\right]}\right)\\ \left[P_{k}\right] & =\left(I-\left[K_{k}\right]\left[H\right]\right)\left[P_{k}^{-}\right] \end{array}\right.$$

However, note that the inversion of the interval matrix in the IKF algorithm is an approximate solution and that it is NP-hard [19], which may negatively affect the possible result and increase computational complexity during the data fusion process. In order to solve these two problems, we propose a new algorithm called the interval split covariance intersection filter (ISCIF), which is based on the SCIF. In the ISCIF, the inversion of the interval covariance matrix is substituted with the ordinary matrix operation, which avoids the stated drawbacks of the IKF.

In the proposed ISCIF, the input estimate to be fused can be denoted as $\left(\left[X\right],P_{I}+P_{D}\right)$, where $\left[X\right]$ is the box state that can be provided by the interval analysis technique, $P_{I}$ is the degree of the independent part of the covariance matrix, and $P_{D}$ is the maximum degree of the part possibly correlated with others. Note that the covariance matrix is divided into independent and correlated parts, which takes advantage of the optimization process of the covariance part in [25]. Given two input estimates to be fused—$\left(\left[A\right],P_{AI}+P_{AD}\right)$ and $\left(\left[B\right],P_{BI}+P_{BD}\right)$—the formula of the ISCIF with the fusion result $\left(\left[C\right],P_{CI}+P_{CD}\right)$ can be represented by:

(15) $$\left\{\begin{array}{cc} P_{1} & =P_{AD}/w+P_{AI}\\ P_{2} & =P_{BD}/\left(1-w\right)+P_{BI}\\ P_{C}^{-1} & =P_{1}^{-1}+P_{2}^{-1}\\ \left[C\right] & =P_{C}\left(P_{1}^{-1}\left[A\right]+P_{2}^{-1}\left[B\right]\right)\\ P_{CI} & =P_{C}\left(P_{1}^{-1}P_{AI}P_{1}^{-1}+P_{2}^{-1}P_{BI}P_{2}^{-1}\right)P_{C}\\ P_{CD} & =I-P_{CI} \end{array}.\right.$$

The weighting coefficient *w* belongs to the interval $\left[0,1\right]$, and it is determined by minimizing the trace or determinant of the output covariance matrix $P_{C}$. In our work, *w* is calculated by minimizing the trace of $P_{C}$. The proposed ISCIF has the same iterative structure with the traditional SCIF, and no additional assumptions are added. It can be used for interval discrete-time systems where the SCIF is not suitable. In addition, when the width of each variable equals zero, the ISCIF will degenerate into SCIF, which increases the applicability of the proposed ISCIF.

### 2.4. Consistency Proof of the Proposed ISCIF

To avoid the over-convergence problem of data fusion results caused by inter-estimate correlation, the data fusion method must be consistent. The consistency of the SCIF with two input resources was reported in [25]. However, it is insufficient for data fusion processes with multiple input resources [26]. So, we give the general proof for the consistency of our proposed ISCIF algorithm, which is also a complementary proof for the SCIF. Note that, in the following proof, the general form of Equation (15), which has *n* data fusion resources $\left\{\left[X_{1}\right],P_{1I}+P_{1D}\right\}$, $\left\{\left[X_{2}\right],P_{2I}+P_{2D}\right\}$, ⋯, $\left\{\left[X_{n}\right],P_{nI}+P_{nD}\right\}$ with the output result $\left\{\left[X\right],P_{I}+P_{D}\right\}$, is used, as shown in Equation (16):

(16) $$\left\{\begin{array}{cc} P_{1} & =\frac{P_{1D}}{w_{1}}+P_{1I}\\ P_{2} & =\frac{P_{2D}}{w_{2}}+P_{2I}\\ \vdots \\ P_{n} & =\frac{P_{nD}}{w_{n}}+P_{nI}\\ P & ={\left(\sum_{k=1}^{n}{P_{k}}^{-1}\right)}^{-1}\\ \left[X\right] & =P(\sum_{k=1}^{n}{P_{k}}^{-1}\left[X_{k}\right])\\ P_{I} & =P(\sum_{k=1}^{n}{P_{k}}^{-1}{P_{kI}}^{-1}{P_{k}}^{-1})P\\ P_{D} & =I-P_{I} \end{array},\right.$$

where the $w_{k}$ satisfies $\sum_{k=1}^{n}w_{k}=1$, and $w_{k}\in \left[0,1\right]$. In addition, the definition of the split form consistency has been reported in [25]. It always satisfies the consistency definition of the KF, and it includes A-split and B-split forms.

For the estimate in split form, it is A-split consistent if it satisfies

(17) $$\begin{array}{cc} P_{D}\geq P_{D}^{*} & =E\left[\bar{X_{D}}\times {\bar{X_{D}}}^{T}\right]\\ P_{I}\geq P_{I}^{*} & =E\left[\bar{X_{I}}\times {\bar{X_{I}}}^{T}\right]. \end{array}$$

It is B-split consistent if it satisfies

(18) $$\begin{array}{cc} P_{D}\geq P_{D}^{*} & =E\left[\bar{X_{D}}\times {\bar{X_{D}}}^{T}\right]\\ P_{I}+P_{D}\geq P_{I}^{*}+P_{D}^{*} & =E\left[\bar{X}\times {\bar{X}}^{T}\right], \end{array}$$

where $\bar{X_{D}}$, $\bar{X_{I}}$, and $\bar{X}$ are the estimated error of the correlated, independent, and whole parts, respectively. In the ISCIF, the estimated error can be determined by calculating the difference between the midpoint of the estimated intervals and the true values.

**Theorem 1.**  *If the n source estimates are A-split consistent, for every $w_{n}\in \left[0,1\right]$, the data fusion result of ISCIF is consistent.*

**Proof of Theorem 1.**  Since the *n* (n≥2) source estimates are A-split consistent, we obtain:

(19) $$\begin{array}{cc} P_{nD}\geq P_{nD}^{*} & =E\left[\bar{X_{nD}}\times {\bar{X_{nD}}}^{T}\right]\\ P_{nI}\geq P_{nI}^{*} & =E\left[\bar{X_{nI}}\times {\bar{X_{nI}}}^{T}\right]. \end{array}$$

To begin with, the proof of the independent component of the fusion result can be given as follows:

(20) $$\begin{array}{c} P_{I}=P\left(P_{1}^{-1}P_{1I}P_{1}^{-1}+\cdots +P_{n}^{-1}P_{nI}P_{n}^{-1}\right)P \end{array}$$

(21) $$\;\begin{array}{c} \bar{X_{I}}=P\left(P_{1}^{-1}\bar{X_{1I}}+\cdots +P_{n}^{-1}\bar{X_{nI}}\right) \end{array}$$

(22) $$\begin{array}{cc} P_{I}-P_{I}^{*} & =P_{I}-E\left[\bar{X_{I}}\times {\bar{X_{I}}}^{T}\right]\\ \; & =\sum_{k=1}^{n}P\left(P_{k}^{-1}P_{kI}P_{k}^{-1}\right)P-\sum_{k=1}^{n}PP_{k}^{-1}E\left[\bar{X_{kI}}\times {\bar{X_{kI}}}^{T}\right]P_{k}^{-1}P\\ \; & =\sum_{k=1}^{n}PP_{k}^{-1}\left(P_{kI}^{-1}-E\left[\bar{X_{kI}}\times {\bar{X_{kI}}}^{T}\right]\right)P_{k}^{-1}P\\ \; & \geq 0. \end{array}$$

The proof of the independent component of the fusion result is finished. Then, the proof of the correlated component of the fusion result is shown as follows:

(23) $$\begin{array}{c} \begin{array}{cc} P_{D} & =P-P_{I}\\ \; & =P-\sum_{k=1}^{n}PP_{k}^{-1}P_{kI}P_{k}^{-1}P\\ \; & =P\left[P^{-1}-\sum_{k=1}^{n}\left(P_{k}^{-1}P_{kI}P_{k}^{-1}\right)\right]P\\ \; & =P\left[\sum_{k=1}^{n}P_{k}^{-1}\left(P_{k}-P_{kI}\right)P_{k}^{-1}\right]P\\ \; & =\sum_{k=1}^{n}PP_{k}^{-1}\frac{P_{kD}}{w_{k}}P_{k}^{-1}P\\ \; & \geq \sum_{k=1}^{n}PP_{k}^{-1}\frac{E\left[\bar{X_{kD}}\times {\bar{X_{kD}}}^{T}\right]}{w_{k}}P_{k}^{-1}P. \end{array} \end{array}$$

The correlated estimated error satisfies:

(24) $$\bar{X_{D}}=\sum_{k=1}^{n}PP_{k}^{-1}\bar{X_{kD}}.$$

So:

(25) $$\begin{array}{cc} \; & P_{D}-E\left[\bar{X_{D}}\times {\bar{X_{D}}}^{T}\right]\\ \; & \geq \sum_{k=1}^{n}PP_{k}^{-1}\frac{E\left[\bar{X_{kD}}\times {\bar{X_{kD}}}^{T}\right]}{w_{k}}P_{k}^{-1}P\\ \; & -P\left\{\sum_{k=1}^{n}\sum_{m=1}^{n}P_{k}^{-1}E\left[\bar{X_{kD}}\times {\bar{X_{mD}}}^{T}\right]P_{m}^{-1}\right\}P\\ \; & =\sum_{k=1}^{n}PP_{k}^{-1}\frac{1-w_{k}}{w_{k}}E\left[\bar{X_{kD}}\times {\bar{X_{kD}}}^{T}\right]P_{k}^{-1}P\\ \; & -\sum_{k=1}^{n}\sum_{m=1,m\neq k}^{n}PP_{k}^{-1}E\left[\bar{X_{kD}}\times {\bar{X_{mD}}}^{T}\right]P_{m}^{-1}P. \end{array}$$

Note that:

(26) $$\sum_{m=1,m\neq k}^{n}w_{m}=1-w_{k}$$

(27) $$\begin{array}{c} \begin{array}{cc} \; & P_{D}-E\left[\bar{X_{D}}\times {\bar{X_{D}}}^{T}\right]\geq \\ \; & \sum_{k=1}^{n}\sum_{m=k+1}^{n}\frac{w_{m}}{w_{k}}PP_{k}^{-1}E\left[\bar{X_{kD}}\times {\bar{X_{kD}}}^{T}\right]P_{k}^{-1}P\\ \; & +\sum_{k=1}^{n}\sum_{m=k+1}^{n}\frac{w_{k}}{w_{m}}PP_{m}^{-1}E\left[\bar{X_{mD}}\times {\bar{X_{mD}}}^{T}\right]P_{m}^{-1}P\\ \; & -\sum_{k=1}^{n}\sum_{m=k+1}^{n}PP_{k}^{-1}E\left[\bar{X_{kD}}\times {\bar{X_{mD}}}^{T}\right]P_{m}^{-1}P\\ \; & -\sum_{k=1}^{n}\sum_{m=k+1}^{n}PP_{m}^{-1}E\left[\bar{X_{mD}}\times {\bar{X_{kD}}}^{T}\right]P_{k}^{-1}P\\ \; & =\sum_{k=1}^{n}\sum_{m=k+1}^{n}\frac{w_{m}^{2}}{w_{k}w_{m}}PP_{k}^{-1}E\left[\bar{X_{kD}}\times {\bar{X_{kD}}}^{T}\right]P_{k}^{-1}P\\ \; & +\sum_{k=1}^{n}\sum_{m=k+1}^{n}\frac{w_{k}^{2}}{w_{k}w_{m}}PP_{m}^{-1}E\left[\bar{X_{mD}}\times {\bar{X_{mD}}}^{T}\right]P_{m}^{-1}P\\ \; & -\sum_{k=1}^{n}\sum_{m=k+1}^{n}\frac{w_{k}w_{m}}{w_{k}w_{m}}PP_{k}^{-1}E\left[\bar{X_{kD}}\times {\bar{X_{mD}}}^{T}\right]P_{m}^{-1}P\\ \; & -\sum_{k=1}^{n}\sum_{m=k+1}^{n}\frac{w_{k}w_{m}}{w_{m}w_{k}}PP_{m}^{-1}E\left[\bar{X_{mD}}\times {\bar{X_{kD}}}^{T}\right]P_{k}^{-1}P\\ \; & =P\left\{\sum_{k=1}^{n}\sum_{m=k+1}^{n}\frac{1}{w_{k}w_{m}}E\left[A\times A^{T}\right]\right\}P, \end{array} \end{array}$$

where $A=\left(w_{m}P_{k}^{-1}\bar{X_{kD}}-w_{k}P_{m}^{-1}\bar{X_{mD}}\right)$. Since $w_{k}$ and $w_{m}$ both belong to the interval $\left[0,1\right]$, therefore:

(28) $$P_{D}-E\left[\bar{X_{D}}\times {\bar{X_{D}}}^{T}\right]\geq 0.$$

The proof of Theorem 1 is finished. □

**Theorem 2.**  *If the n source estimates are B-split consistent, for each $w_{n}\in \left[0,1\right]$, the data fusion result of ISCIF is consistent.*

**Proof of Theorem 2.**  Based on the definition of the B-split consistency, we can obtain:

(29) $$\begin{array}{cc} \; & P_{nD}\geq E\left[\bar{X_{nD}}\times {\bar{X_{nD}}}^{T}\right]\\ \; & P_{nD}+P_{nI}\geq E\left[\bar{X_{n}}\times {\bar{X_{n}}}^{T}\right]. \end{array}$$

Note that the B-split consistency of the correlated part $P_{D}$ can be proven identically as in Theorem 1. The proof of the entire covariance *P* is shown as follows:

(30) $$\begin{array}{cc} \; & P=P_{D}+P_{I}\\ \; & =P\left\{\sum_{k=1}^{n}P_{k}^{-1}P_{k}P_{k}^{-1}\right\}P\\ \; & =P\left\{\sum_{k=1}^{n}P_{k}^{-1}\left(\frac{P_{kD}}{w_{k}}+P_{kI}\right)P_{k}^{-1}\right\}P\\ \; & =\sum_{k=1}^{n}PP_{k}^{-1}P_{kI}P_{k}^{-1}P\\ \; & +\sum_{k=1}^{n}PP_{k}^{-1}\left(\frac{P_{kD}-E\left[\bar{X_{kD}}\times {\bar{X_{kD}}}^{T}\right]}{w_{k}}\right)P_{k}^{-1}P\\ \; & +\sum_{k=1}^{n}PP_{k}^{-1}\left(\frac{E\left[\bar{X_{kD}}\times {\bar{X_{kD}}}^{T}\right]}{w_{k}}\right)P_{k}^{-1}P\\ \; & \geq \sum_{k=1}^{n}PP_{k}^{-1}E\left[\bar{X_{kI}}\times {\bar{X_{kI}}}^{T}\right]P_{k}^{-1}P\\ \; & +\sum_{k=1}^{n}PP_{k}^{-1}\frac{E\left[\bar{X_{kD}}\times {\bar{X_{kD}}}^{T}\right]}{w_{k}}P_{k}^{-1}P \end{array}$$

(31) $$\begin{array}{cc} \; & {\bar{X}}_{I}=\sum_{k=1}^{n}P\left(P_{k}^{-1}\bar{X_{kI}}\right)\\ \; & {\bar{X}}_{D}=\sum_{k=1}^{n}P\left(P_{k}^{-1}\bar{X_{kD}}\right)\\ \; & E\left[\bar{X}\times {\bar{X}}^{T}\right]=E\left[\bar{X_{I}}\times {\bar{X_{I}}}^{T}\right]+E\left[\bar{X_{D}}\times {\bar{X_{D}}}^{T}\right] \end{array}$$

(32) $$\begin{array}{cc} \; & P-E\left[\bar{X}\times {\bar{X}}^{T}\right]\\ \; & =P_{I}+P_{D}-E\left[\bar{X}\times {\bar{X}}^{T}\right]\\ \; & \geq \sum_{k=1}^{n}PP_{k}^{-1}E\left[\bar{X_{kI}}\times {\bar{X_{kI}}}^{T}\right]P_{k}^{-1}P\\ \; & +\sum_{k=1}^{n}PP_{k}^{-1}\frac{E\left[\bar{X_{kD}}\times {\bar{X_{kD}}}^{T}\right]}{w_{k}}P_{k}^{-1}P\\ \; & -\sum_{k=1}^{n}PP_{k}^{-1}E\left[\bar{X_{kI}}\times {\bar{X_{kI}}}^{T}\right]P_{k}^{-1}P\\ \; & -\sum_{k=1}^{n}\sum_{m=1}^{n}PP_{k}^{-1}E\left[\bar{X_{kD}}\times {\bar{X_{mD}}}^{T}\right]P_{m}^{-1}P\\ \; & =\sum_{k=1}^{n}PP_{k}^{-1}\frac{1-w_{k}}{w_{k}}E\left[\bar{X_{kD}}\times {\bar{X_{kD}}}^{T}\right]P_{k}^{-1}P\\ \; & -\sum_{k=1}^{n}\sum_{m=1,m\neq k}^{n}PP_{k}^{-1}E\left[\bar{X_{kD}}\times {\bar{X_{mD}}}^{T}\right]P_{m}^{-1}P. \end{array}$$

Note that the result of Equation (32) is the same as that in Equation (25). Since $\sum_{m=1,m\neq k}^{n}w_{m}=1-w_{k}$, combining it with Equation (27), the entire covariance *P* satisfies:

(33) $$\begin{array}{cc} \; & P-E\left[\bar{X}\times {\bar{X}}^{T}\right]\geq 0. \end{array}$$

Meanwhile, the proof of Theorem 2 is finished. Note that, when the number of data fusion resources satisfies n=2, the general proof of the ISCIF is the same as that in [25]. Given the general proofs of both A-split and B-split consistencies above, either the A-split or B-split consistency can guarantee the consistency of the data fusion result, which means that it is not necessary to always distinguish or give the explicit definition before using the proposed algorithm. Furthermore, no additional confidence is incorporated into the data fusion process, thus mitigating the issue of over-convergence stemming from correlated data. □

## 3. MSMV Cooperative Vehicle Localization

### 3.1. System Model

**Figure 4:**
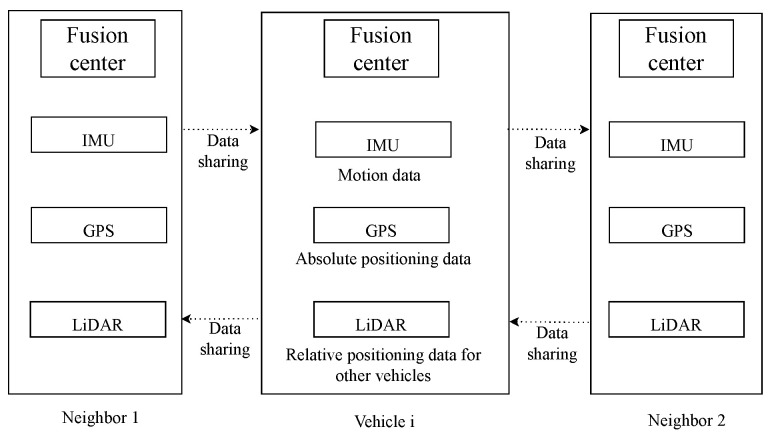
Decentralized cooperative localization architecture [15].

We consider the general system model that consists of multiple sensors and multiple vehicles, where the number of vehicles, *N*, is unknown, as presented in [20]. The following assumptions are met by our system model:

1. Each vehicle is equipped with an IMU, GPS, and LiDAR, which respectively provide motion data, absolute positioning data, and relative positioning data.
2. When vehicles are within communication range, data sharing is realized by the V2V communication technique.
3. The designed cooperative vehicle localization system works in a decentralized manner, which is shown in Figure 4. In addition, each vehicle can timestamp its data using global system time, which can increase the real-time performance of the localization system.

### 3.2. Prediction of the State Vector

In our model, the state vector or box of each vehicle includes the position in terms of both the *x* and *y* coordinates, as well as the orientation of the vehicles. The state vector of vehicle *i* at time *t* is represented as follows:

(34) $$X_{i}\left(t\right)={\left[x_{i}\left(t\right),y_{i}\left(t\right),\theta_{i}\left(t\right)\right]}^{T}.$$

The coordinates of the vehicle on the *x*-axis and *y*-axis are, respectively, $x_{i}\left(t\right)$ and $y_{i}\left(t\right)$, and $\theta_{i}\left(t\right)$ is the direction of the vehicle.

Using the kinematic bicycle model, the propagation model is defined as:

(35) $$\left\{\begin{array}{c} x_{i}\left(t\right)=x_{i}\left(t-1\right)+\Delta d_{i}\left(t\right)cos\left(\theta_{i}\left(t-1\right)+\frac{\Delta \theta_{i}\left(t\right)}{2}\right)\\ y_{i}\left(t\right)=y_{i}\left(t-1\right)+\Delta d_{i}\left(t\right)sin\left(\theta_{i}\left(t-1\right)+\frac{\Delta \theta_{i}\left(t\right)}{2}\right)\\ \theta_{i}\left(t\right)=\theta_{i}\left(t-1\right)+\Delta \theta_{i}\left(t\right) \end{array}\right.,$$

where $\Delta d_{i}\left(t\right)$ is the distance the vehicle traveled from time t−1 to time *t*, and $\Delta \theta_{i}\left(t\right)$ is the difference in the directional angle of the vehicle from time t−1 to time *t*.

Adding the process noise, the propagation model can be expressed in the function form as follows:

(36) $$\left[\begin{array}{c} x_{i}\left(t\right)\\ y_{i}\left(t\right)\\ \theta_{i}\left(t\right) \end{array}\right]\;=\;f\left(\left[\begin{array}{c} x_{i}\left(t-1\right)\\ y_{i}\left(t-1\right)\\ \theta_{i}\left(t-1\right) \end{array}\right],\left[\begin{array}{c} \Delta d_{i}\left(t\right)\\ \Delta \theta_{i}\left(t\right) \end{array}\right]\right)+Q_{i}(t),$$

where $Q_{i}\left(t\right)$ is the Gaussian white noise of the process state. We assume that the noise of the motion measurements follows a Gaussian distribution with zero mean, and the covariance matrix is $Q_{i}^{U}\left(t\right)$. So, the $Q_{i}^{U}\left(t\right)$ can be defined as follows:

(37) $$Q_{i}^{U}\left(t\right)=\left[\begin{array}{cc} \sigma_{\Delta d_{i}\left(t\right)}^{2} & 0\\ 0 & \sigma_{\Delta \theta_{i}\left(t\right)}^{2} \end{array}\right].$$

The covariance of vehicle *i* at time *t* can be updated by the following:

(38) $$\begin{array}{cc} \; & P_{i}\left(t\right)=F_{i}\left(t\right)P_{i}\left(t-1\right)F_{i}{\left(t\right)}^{T}\\ \; & +B_{i}\left(t\right)Q_{i}^{U}\left(t\right)B_{i}{\left(t\right)}^{T}+Q_{i}\left(t\right), \end{array}$$

where $F_{i}\left(t\right)$ and $B_{i}\left(t\right)$ are Jacobian matrices that are calculated by the following equations:

(39) $$F_{i}\left(t\right)=\left[\begin{array}{ccc} 1 & 0 & -\Delta d_{i}\left(t\right)sin\left\{\theta_{i}\left(t-1\right)+\frac{\Delta \theta_{i}\left(t\right)}{2}\right\}\\ 0 & 1 & \Delta d_{i}\left(t\right)cos\left\{\theta_{i}\left(t-1\right)+\frac{\Delta \theta_{i}\left(t\right)}{2}\right\}\\ 0 & 0 & 1 \end{array}\right]$$

(40) $$\begin{array}{cc} \; & B_{i}\left(t\right)=\left[\begin{array}{cc} cos\left\{\theta_{i}\left(t-1\right)+\frac{\Delta \theta_{i}\left(t\right)}{2}\right\} & -\frac{1}{2}\Delta d_{i}\left(t\right)sin\left\{\theta_{i}\left(t-1\right)+\frac{\Delta \theta_{i}\left(t\right)}{2}\right\}\\ sin\left\{\theta_{i}\left(t-1\right)+\frac{\Delta \theta_{i}\left(t\right)}{2}\right\} & \frac{1}{2}cos\left\{\theta_{i}\left(t-1\right)+\frac{\Delta \theta_{i}\left(t\right)}{2}\right\}\\ 0 & 1 \end{array}\right]. \end{array}$$

Since both the total state covariance $P_{i}\left(t\right)$ and the independent part $P_{iI}\left(t\right)$ can be determined at each time, the independent part is calculated by

(41) $$\begin{array}{cc} \; & P_{iI}\left(t\right)=F_{i}\left(t\right)P_{iI}\left(t-1\right)F_{i}{\left(t\right)}^{T}\\ \; & +B_{i}\left(t\right)Q_{i}^{U}\left(t\right)B_{i}{\left(t\right)}^{T}+Q_{iI}\left(t\right), \end{array}$$

where $Q_{iI}\left(t\right)$ is the independent component of $Q_{i}\left(t\right)$.

### 3.3. Absolute Positioning Stage

Since the absolute localization measurement provided by the GPS at each time is completely independent of any existing estimates or measurements, there is no data incest problem in the absolute positioning stage. The state update with the absolute localization measurement using ISCIF can be degenerated and transferred to the update strategy proposed in [14]. For each vehicle *i*, the absolute measurement provided by GPS can be denoted as $Z_{i}\left(t\right)=\left(x_{i}^{G},y_{i}^{G}\right)$. The observation model of the absolute measurement at time *t* can be represented by

(42) $$Z_{i}\left(t\right)=H_{i}\left(t\right)X_{i}\left(t\right)+R_{i}\left(t\right),$$

where

(43) $$H_{i}\left(t\right)=\left[\begin{array}{ccc} 1 & 0 & 0\\ 0 & 1 & 0 \end{array}\right].$$

Therefore, $R_{i}\left(t\right)$ is the Gaussian white noise, which follows $N\left(0,\sigma_{i}{\left(t\right)}^{2}\right)$. So, the state updates from using the absolute localization measurement (GPS data in our model) are represented in the following equations:

(44) $$\begin{array}{cc} K & =P_{i}\left(t\right)H_{i}^{T}{\left(H_{i}P_{i}\left(t\right)H_{i}^{T}+\sigma_{i}{\left(t\right)}^{2}\right)}^{-1}\\ X_{i}\left(t\right) & =X_{i}\left(t\right)+K\left(Z_{i}\left(t\right)-H_{i}X_{i}\left(t\right)\right)\\ P_{i}\left(t\right) & =\left(I-KH_{i}\right)P_{i}\left(t\right)\\ P_{iI}\left(t\right) & =\left(I-KH_{i}\right)P_{iI}\left(t\right){\left(I-KH_{i}\right)}^{T}+K\sigma_{i}{\left(t\right)}^{2}K^{T}\\ P_{iD}\left(t\right) & =P_{i}\left(t\right)-P_{iI}\left(t\right). \end{array}$$

### 3.4. Relative Positioning Stage

**Figure 5:**
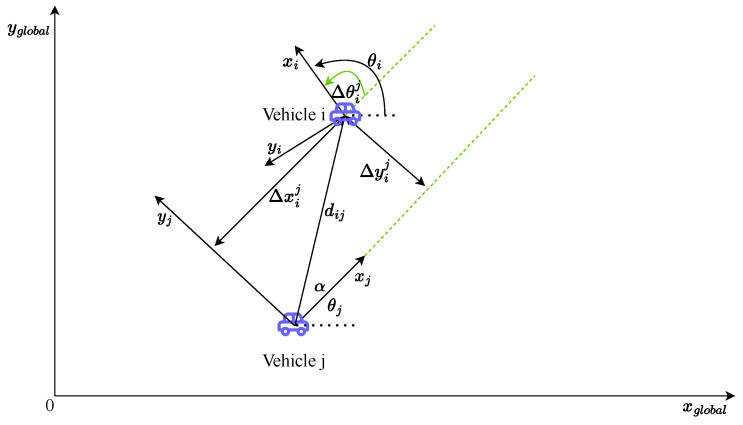
Relative observation between vehicles [15].

After the absolute positioning stage, we use the interval analysis technique to generate the interval (or box) form of the data in the system. The relative observation between two vehicles is shown in Figure 5. When vehicle *i* is observed by vehicle *j*, the relative estimate of vehicle *i* provided by vehicle *j* can be denoted as follows:

(45) $$\left[X_{i}^{j}\left(t\right)\right]=\left[\begin{array}{ccc} \left[cos\right](\left[\theta_{j}\left(t\right)\right]) & -\left[sin\right](\left[\theta_{j}\left(t\right)\right]) & 0\\ \left[sin\right](\left[\theta_{j}\left(t\right)\right]) & \left[cos\right](\left[\theta_{j}\left(t\right)\right]) & 0\\ 0 & 0 & 1 \end{array}\right]\left[\Delta X_{i}^{j}\left(t\right)\right]+\left[X_{j}\left(t\right)\right],$$

where the $\left[X_{i}^{j}\left(t\right)\right]$ represents the box relative estimate of vehicle *i* when it is observed by vehicle *j*; $\left[\Delta X_{i}^{j}\left(t\right)\right]={\left\{\left[\Delta x_{i}^{j}\left(t\right)\right],\left[\Delta y_{i}^{j}\left(t\right)\right],\left[\Delta \theta_{i}^{j}\left(t\right)\right]\right\}}^{T}$ are the relative measurements in the box form; and $\left[X_{j}\left(t\right)\right]={\left\{\left[x_{j}\left(t\right)\right],\left[y_{j}\left(t\right)\right],\left[\theta_{j}\left(t\right)\right]\right\}}^{T}$ is the box state of the vehicle *j* in the global coordinate system.

Based on α(t) and $d_{ij}\left(t\right)$, which are provided by sensors, the interval relative distance $\left[d_{ij}\left(t\right)\right]$ and the interval relative bearing [α(t)] can be calculated by

(46) $$\begin{array}{cc} \left[\alpha (t)\right] & =\left[\alpha \left(t\right)-3\sigma_{\alpha },\alpha \left(t\right)+3\sigma_{\alpha }\right]\\ \left[d_{ij}\left(t\right)\right] & =\left[d_{ij}\left(t\right)-3\sigma_{d},d_{ij}\left(t\right)+3\sigma_{d}\right], \end{array}$$

where $\sigma_{\alpha }$ and $\sigma_{d}$ are the standard deviation of the sensors. So, $\left[\Delta x_{i}^{j}\left(t\right)\right]$ and $\left[\Delta y_{i}^{j}\left(t\right)\right]$ can be calculated by

(47) $$\begin{array}{cc} \; & \left[\Delta x_{i}^{j}\left(t\right)\right]=\left[cos\right]\left(\left[\alpha (t)\right]\right)*\left[d_{ij}\left(t\right)\right]\\ \; & \left[\Delta y_{i}^{j}\left(t\right)\right]=\left[sin\right]\left(\left[\alpha (t)\right]\right)*\left[d_{ij}\left(t\right)\right], \end{array}$$

where [cos] and [sin] represent the inclusion function. In addition, $\left[X_{j}\left(t\right)\right]$ in Equation (45) is optimized by adding tolerance variables ($\alpha_{xj}$, $\alpha_{yj}$, and $\alpha_{\theta j}$) by interval analysis as follows:

(48) $$\begin{array}{cc} \; & \left[x_{j}\left(t\right)\right]=\left[x_{j}\left(t\right)-\alpha_{xj},x_{j}\left(t\right)+\alpha_{xj}\right]\\ \; & \left[y_{j}\left(t\right)\right]=\left[y_{j}\left(t\right)-\alpha_{yj},y_{j}\left(t\right)+\alpha_{yj}\right]\\ \; & \left[\theta_{j}\left(t\right)\right]=\left[\theta_{j}\left(t\right)-\alpha_{\theta j},\theta_{j}\left(t\right)+\alpha_{\theta j}\right], \end{array}$$

where $x_{j}\left(t\right)$, $y_{j}\left(t\right)$, and $\theta_{j}\left(t\right)$ are the result of Equation (44). Meanwhile, regarding the covariance part, we can calculate the covariance matrix of the relative estimate of vehicle *i*, which is provided by vehicle *j*, using the following equations:

(49) $$\begin{array}{cc} \; & P_{X_{i}^{j}}\left(t\right)=J_{1}P_{j}\left(t\right)J_{1}^{T}+J_{2}Q_{j}J_{2}^{T}\\ \; & P_{X_{iI}^{j}}\left(t\right)=J_{1}P_{jI}\left(t\right)J_{1}^{T}+J_{2}Q_{jI}J_{2}^{T}, \end{array}$$

where $P_{X_{i}^{j}}\left(t\right)$ and $P_{X_{iI}^{j}}\left(t\right)$ are the total covariance of the relative estimate and the independent part of covariance at time *t*, respectively; and $P_{j}\left(t\right)$ and $P_{jI}\left(t\right)$ are the total covariances of the state of vehicle *j* in the global coordinate system and the independent part of $P_{j}\left(t\right)$, respectively. In addition, $J_{1}$ and $J_{2}$ are Jacobian matrices with respect to $X_{j}\left(t\right)$ and the relative measurements $\Delta X_{i}^{j}\left(t\right)$, respectively. They can be denoted as follows:

(50) $$\begin{array}{cc} \; & J_{1}=\frac{\partial X_{i}^{j}}{\partial X_{j}\left(t\right)}=\left[\begin{array}{ccc} 1 & 0 & -\Delta x_{i}^{j}sin\theta_{j}-\Delta y_{i}^{j}cos\theta_{j}\\ 0 & 1 & \Delta x_{i}^{j}cos\theta_{j}-\Delta y_{i}^{j}sin\theta_{j}\\ 0 & 0 & 1 \end{array}\right]\\ \; & J_{2}=\frac{\partial X_{i}^{j}}{\partial \Delta X_{i}^{j}\left(t\right)}=\left[\begin{array}{ccc} cos\theta_{j} & -sin\theta_{j} & 0\\ sin\theta_{j} & cos\theta_{j} & 0\\ 0 & 0 & 1 \end{array}\right]. \end{array}$$

Before the state update using ISCIF, we use the ICP method to preprocess multiple relative estimates. The relative estimate result $\left[X_{i}^{R(t)}\right]$ that fuses all relative estimates can be provided by solving the ICSP using the ICP method in Equation (11). Moreover, in order to increase the localization accuracy, we use the same method in Equation (48) using parameters $\beta_{xi}$, $\beta_{yi}$, and $\beta_{\theta i}$ to process the result in the absolute positioning stage (Equation (44)) as follows:

(51) $$\begin{array}{cc} \; & \left[x_{i}\left(t\right)\right]=\left[x_{i}\left(t\right)-\beta_{xi},x_{i}\left(t\right)+\beta_{xi}\right]\\ \; & \left[y_{i}\left(t\right)\right]=\left[y_{i}\left(t\right)-\beta_{yi},y_{i}\left(t\right)+\beta_{yi}\right]\\ \; & \left[\theta_{i}\left(t\right)\right]=\left[\theta_{i}\left(t\right)-\beta_{\theta i},\theta_{i}\left(t\right)+\beta_{\theta i}\right]. \end{array}$$

The box state of vehicle *i* with relative information can be represented as follows:

(52) $$\left\{\left[X_{i}\left(t\right)\right],P_{iI}\left(t\right)+P_{iD}\left(t\right),\left[X_{i}^{R}\left(t\right)\right]\right\},$$

where $\left[X_{i}\left(t\right)\right]$ is the state box of vehicle *i* at time *t*; $P_{iI}\left(t\right)$ and $P_{iD}\left(t\right)$ are the independent component and correlated component of the covariance matrix, respectively; and $\left[X_{i}^{R}\left(t\right)\right]$ is the result of all relative estimates based on the ICP method, which also constitutes the input data of ISCIF.

Regarding the covariance part, since all the relative estimates have been optimized by the ICP technique with the result $\left[X_{i}^{R}\right]$, we can assume that the related covariance of $\left[X_{i}^{R}\right]$ is equal to the matrix with the minimal trace among all $P_{X_{i}^{j}}\left(t\right)$:

(53) $$\begin{array}{cc} \; & P_{X_{iI}^{R}}=P_{X_{iI}^{k}}\\ \; & P_{X_{iD}^{R}}=P_{X_{iD}^{k}}, \end{array}$$

where $P_{X_{iI}^{R}}$ and $P_{X_{iD}^{R}}$ are the independent component and the correlated component of the related covariance of $\left[X_{i}^{R}\right]$. Moreover, vehicle *k* has the smallest trace.

So, the formula for ISCIF for localization using relative estimates can be defined as follows:

(54) $$\begin{array}{cc} P_{1} & =\frac{P_{iD(t)}}{w}+P_{iI}\left(t\right)\\ P_{2} & =\frac{P_{X_{iD}^{R}\left(t\right)}}{1-w}+P_{X_{iI}^{R}}\left(t\right)\\ K & =P_{1}{\left(P_{1}+P_{2}\right)}^{-1}\\ \left[X_{i}\left(t\right)\right] & =\left[X_{i}\left(t\right)\right]+K\left\{\left[X_{i}^{R}\left(t\right)\right]-\left[X_{i}\left(t\right)\right]\right\}\\ P_{i}\left(t\right) & =\left(I-K\right)P_{1}\\ P_{iI}\left(t\right) & =\left(I-K\right)P_{iI}{\left(I-K\right)}^{T}+KP_{X_{iI}^{R}}\left(t\right)K^{T}\\ P_{iD}\left(t\right) & =P_{i}\left(t\right)-P_{iI}\left(t\right). \end{array}$$

In order to form a loop with localization process of state prediction and absolute positioning stages, the input variables in the state vector $X_{i}\left(t\right)$ (Equation (35)) are determined by calculating the midpoint of the variables in state box $\left[X_{i}\left(t\right)\right]$ (the result in Equation (54)), where the formulation of calculating the midpoint is shown in Equation (3).

### 3.5. Discussion

**Figure 6:**
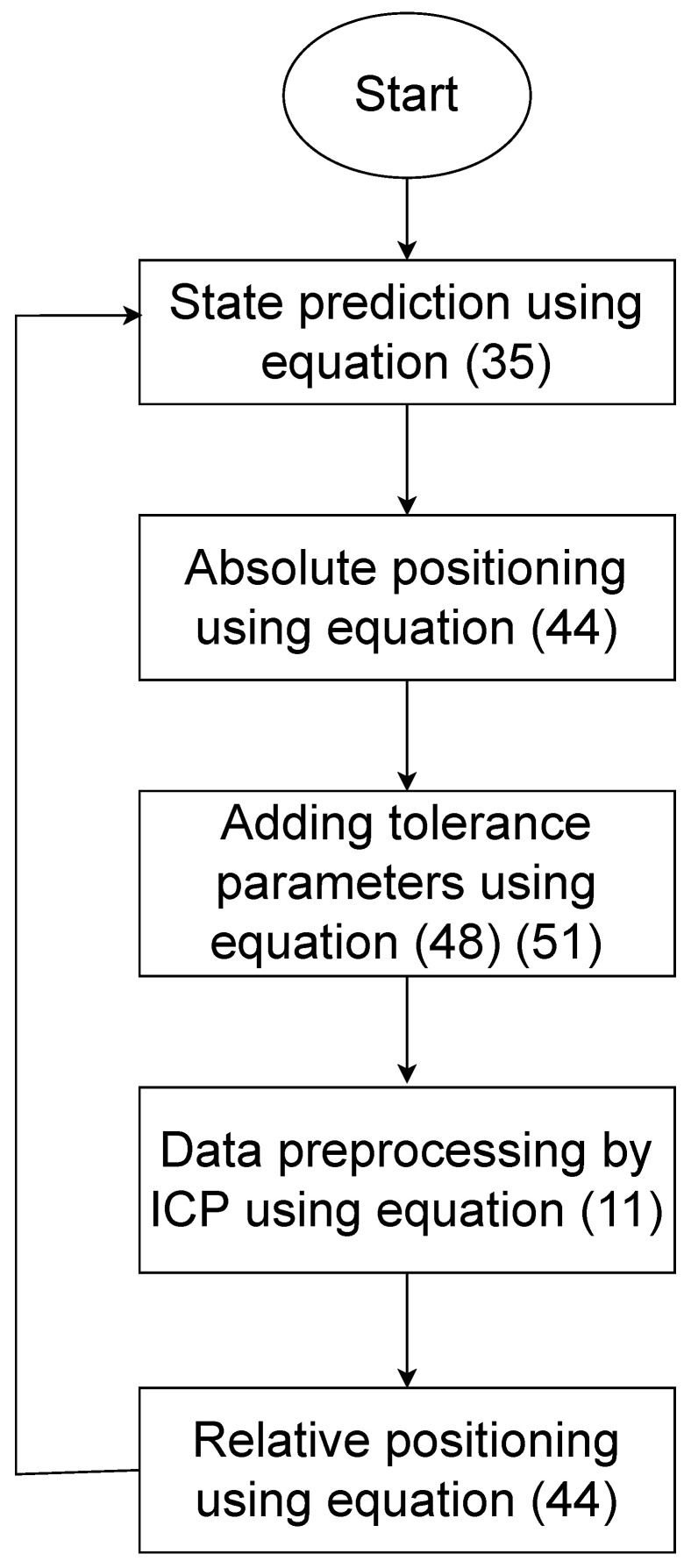
Data process for each vehicle [15].

The localization procedures of each vehicle are illustrated in Figure 6. By using the V2V technique, data sharing can be realized after the absolute positioning stage of each vehicle. Additionally, after the relative positioning stage, the output is in the form of a box. In the prediction stage for the next time step, the input data can be generated using the midpoint of each component within the output box from the last time step. This process provides the iteration for the algorithm.

As mentioned previously, our proposed ISCIF algorithm can be effectively applied to cooperative vehicle localization systems using both absolute and multi-relative measurements. This approach enhances localization accuracy and addresses the issues (e.g., data incest) arising from multiple relative estimates. Additionally, the parameters for determining the interval width in Equation (48) should be adjusted according to the specific requirements of different applications.

Furthermore, considering the communication time delay in V2V communication, the error caused by the time delay can be mitigated by adjusting the noise component in the prediction and update steps [14].

## 4. Simulation Results

### 4.1. Simulation-Based Comparative Study

Simulation experiments are executed to test and compare the performance of our proposed method and several other methods. These methods are introduced as follows:

1. Single vehicle localization (SL) method.
  Each vehicle in the system achieves vehicle localization through the collected motion and GPS data by its own onboard sensors. No cooperation between vehicles is required, and the data fusion method is shown in Equation (44).
2. The ICP method proposed in [22].
  ICP method is used to obtain the prediction result, absolute position measurement, and relative position estimations to achieve vehicle localization, as shown in Equation (10).
3. The SCIF method proposed in [14].
  The SCIF algorithm is employed in both the absolute and relative positioning stages. During the absolute positioning stage, each vehicle updates its own position based on absolute measurements. In the relative positioning stage, when each vehicle receives relative position measurements from neighboring vehicles via the V2V technique, the state vector of each vehicle is updated.
4. Cooperative localization by using ISCIF incorporating ICP (ICP+ISCIF).
  Details have been described previously.

In our proposed method, the ICP method is used to reduce the redundancy of multiple relative estimates, and the ISCIF is employed to perform data fusion in both absolute and relative positioning stages.

Both our proposed method and the ICP method can provide estimated results in interval (or box) form, containing upper and lower bounds as well as midpoints at each time step. According to [21], the midpoint of the estimated interval at each time can be used to demonstrate the positioning accuracy performance. So, the root mean square error (RMSE) or positioning error of our method and the ICP method are calculated based on the midpoint. Moreover, the dataset for the simulation is generated by adding Gaussian noise to both the absolute and relative measurements, based on the true value of each time step.

### 4.2. Simulation Scenario and Parameters

**Figure 7:**
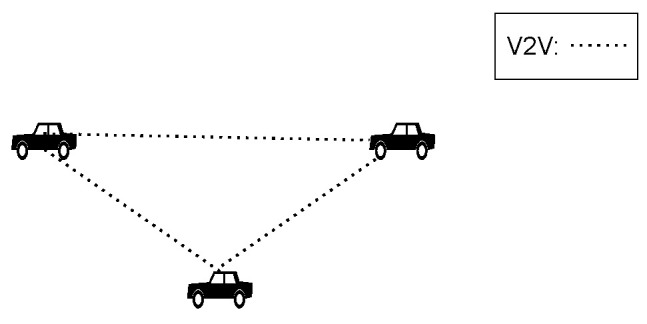
Simulation scenario.

The comparison of the positioning performance of different techniques is based on the scenario illustrated in Figure 7. Three vehicles are capable of observing each other, and data sharing is facilitated by the V2V communication technique. In our simulation scenario, in contrast to that in [14], heightened mutual observation among vehicles exacerbates the data incest problem, as depicted in Figure 1. Therefore, we can more conveniently study the effectiveness of our proposed method in addressing the data incest problem.

Two simulation scenarios are designed to evaluate the localization accuracy of our proposed method. In the first scenario, all vehicles possess identical absolute localization abilities. In the second scenario, the first vehicle has good absolute localization ability, while the remaining vehicles maintain the same absolute positioning capability as the vehicle in the first scenario. All parameters for simulation are illustrated in Table 1.

**Figure 8:**
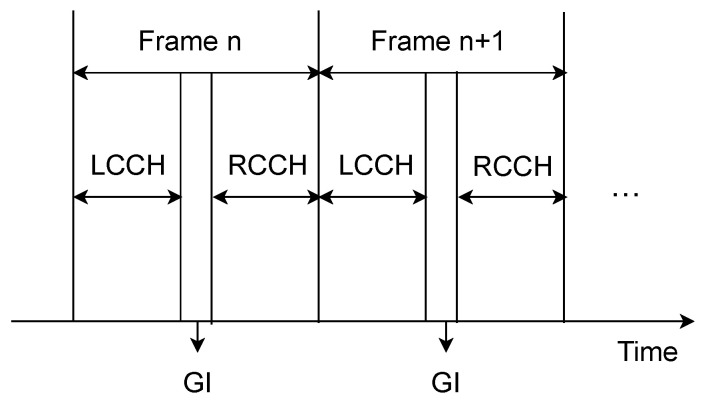
The structure of the TDMA frame.

In addition, the network frequency band for dedicated short-range communication wireless access in vehicular environments is 5.9 GHz. To achieve safer and more effective information sharing between vehicles, we employ a synchronous time division multiple access (TDMA) technique. We adopt the TDMA frame structure outlined in [27], as depicted in Figure 8. Every frame spans 100 ms, during which the left control channel (LCCH) and right control channel (RCCH) are employed for secure data transmission, each enduring for 46 ms. Moreover, a 4 ms guard interval (GI) is included to assist in system synchronization.

### 4.3. All Vehicles Have the Same Absolute Positioning Ability

Simulation 1 is conducted with each vehicle having the same absolute positioning ability; the proposed method and several other methods are executed simultaneously using the same synthetic data.

**Figure 9:**
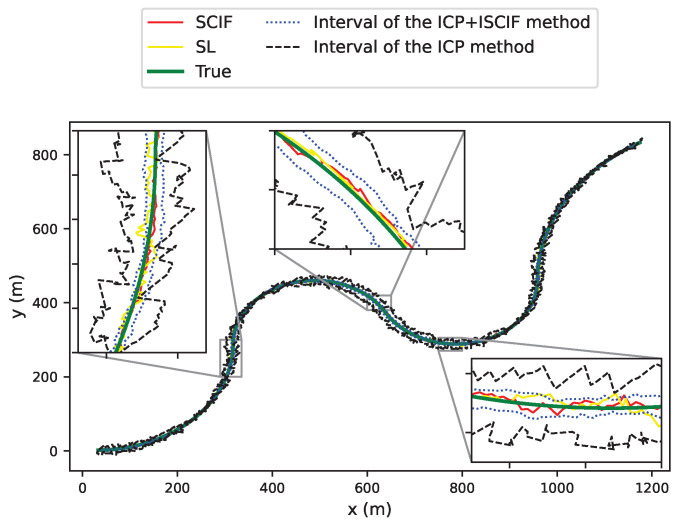
Estimated trajectories for different methods in Scenario 1.

Figure 9 displays the trajectories of Vehicle 1 using different methods over a span of 2 min. Note that both our method and the ICP method can consistently provide an interval that includes the true position of the vehicle almost all the time.

**Figure 10:**
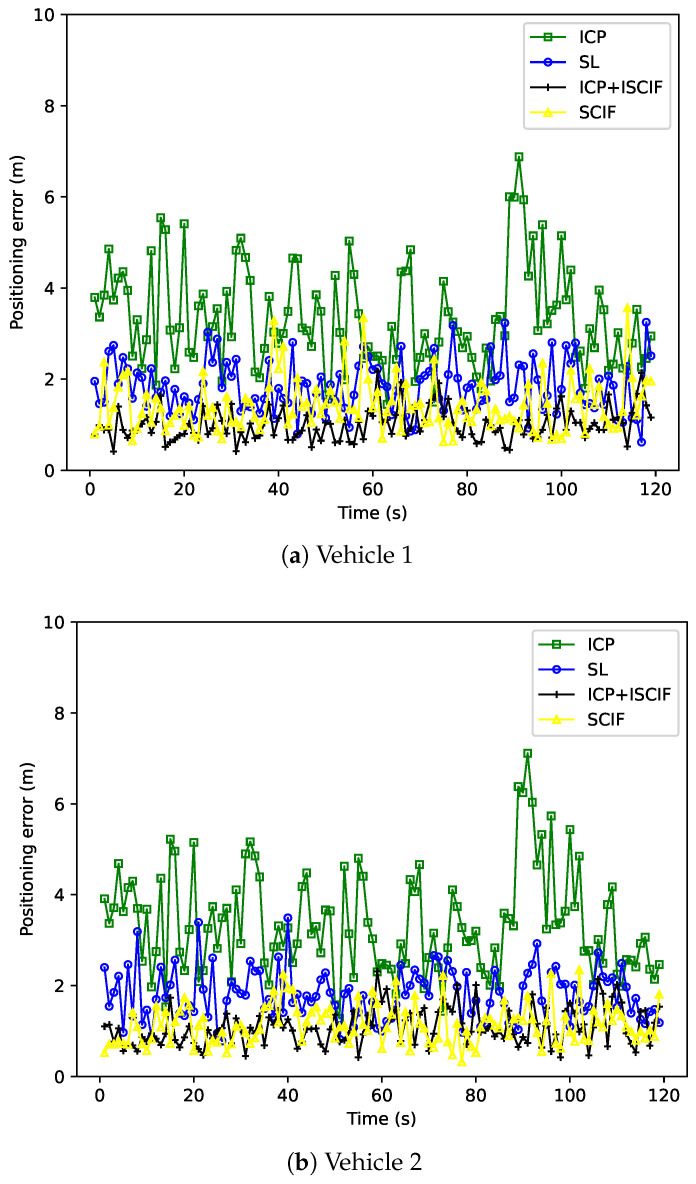

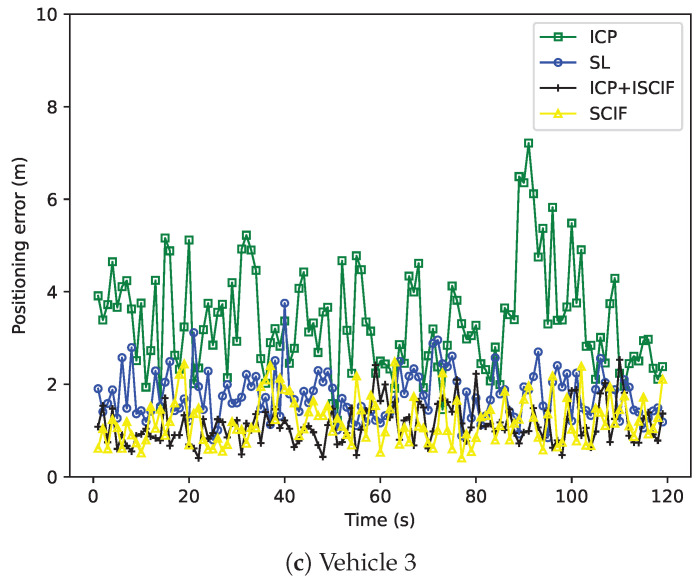
Performance of different methods (with the same absolute positioning ability).

**Figure 11:**
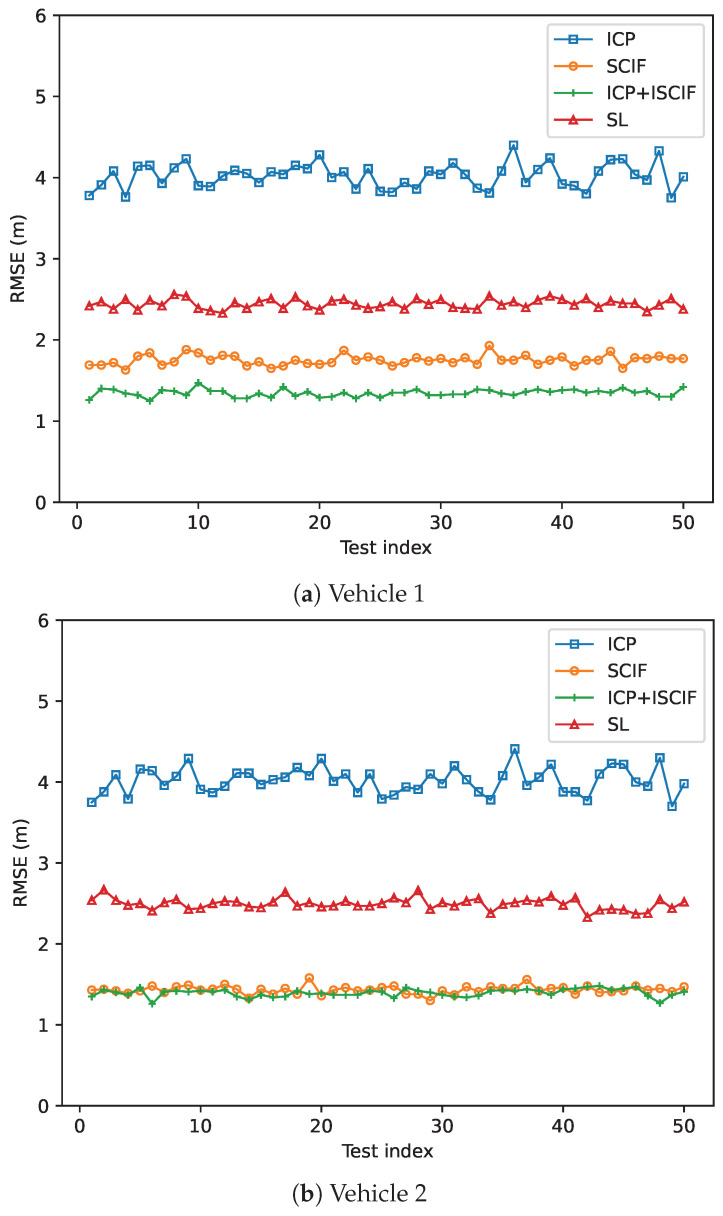

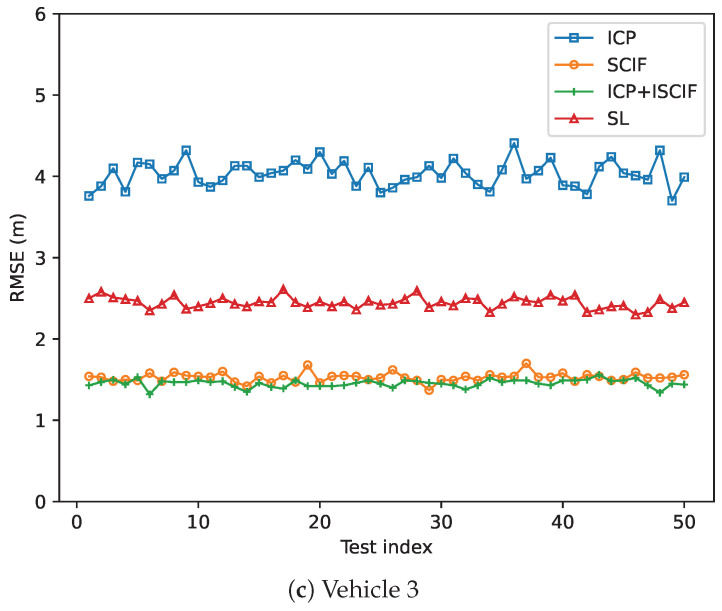
RMSE of each vehicle associated with different methods (with the same absolute positioning ability).

The accuracy performance of each vehicle based on one round of simulation is shown in Figure 10. Each subfigure in Figure 10 represents the positioning error of one vehicle by using different methods in each second. The *x*-axis represents the time sequence, and the positioning error is indicated by the *y*-axis. It is noteworthy that our method achieves the most accurate results compared to the other three methods, demonstrating its effectiveness in solving the data incest problem when facing multiple relative estimates in MSMVs. Furthermore, the SCIF method exhibits better accuracy performance than the SL method, owing to the cooperation between vehicles. Additionally, Figure 11 illustrates the RMSE of 50 rounds of simulation. The *x*-axis denotes the test index, and the RMSE is indicated by the *y*-axis. The average RMSE of three vehicles in 50 tests using the ICP, SL, SCIF, and our method is 4.01 m, 2.46 m, 1.57 m, and 1.40 m, respectively. Our method reduces the RMSE by about 11% compared to the SCIF method.

### 4.4. One Vehicle Has Excellent Absolute Positioning Ability

**Figure 12:**
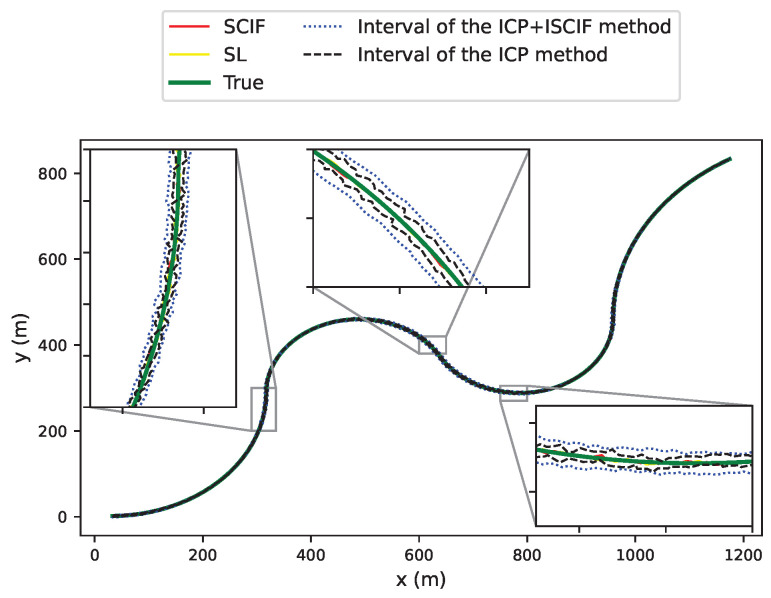
Estimated trajectories for different methods in Scenario 2.

In Scenario 2, the absolute positioning standard errors of Vehicle 1 on both the *x* and *y* coordinates are 0.5 m, while the absolute positioning ability of other vehicles remains unchanged. The trajectories of Vehicle 1 using different methods based on one round of simulation are shown in Figure 12. Note that the true position may not be included in the intervals provided by the ICP method. In other words, the ICP method may lose the possible solution.

**Figure 13:**
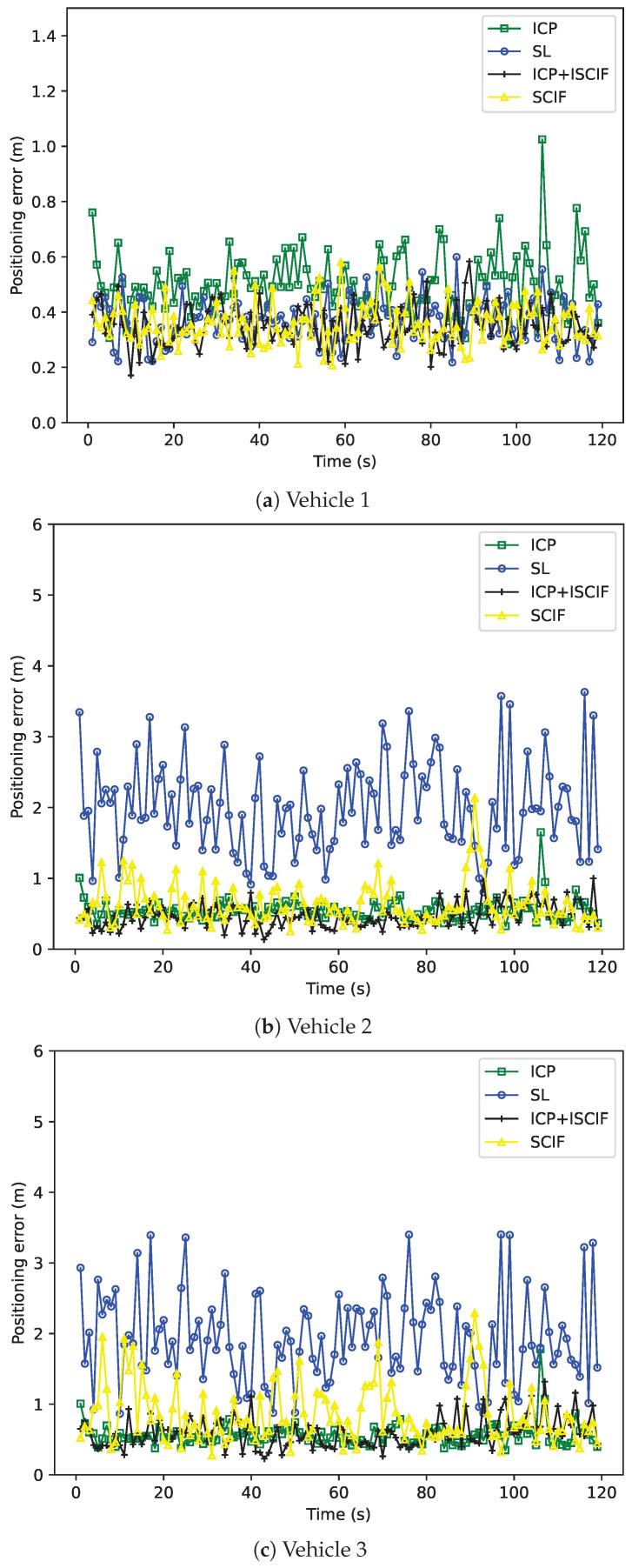
Positioning error of vehicles associated with different methods (Vehicle 1 has excellent positioning ability).

Figure 13 illustrates the comparison results of four methods based on one round of simulation. The *x*-axis in each subfigure represents the time sequence, and the *y*-axis indicates the positioning error. We note that, for all the vehicles, the positioning error provided by the ICP method decreases significantly compared with the results in Figure 10. Additionally, for Vehicle 1, the positioning errors based on the SL, SCIF, and the ICP+ISCIF methods are similar, as the data fusion method utilizing a GPS sensor with excellent absolute positioning ability can achieve reasonable accuracy. Furthermore, for Vehicles 2 and 3, the positioning accuracy based on the ICP, SCIF, and the ICP+ISCIF methods can also be improved compared to that in Scenario 1, benefiting from the mutual cooperation between vehicles. In other words, the cooperative localization method can effectively enhance the accuracy performance compared to the SL method.

**Figure 14:**
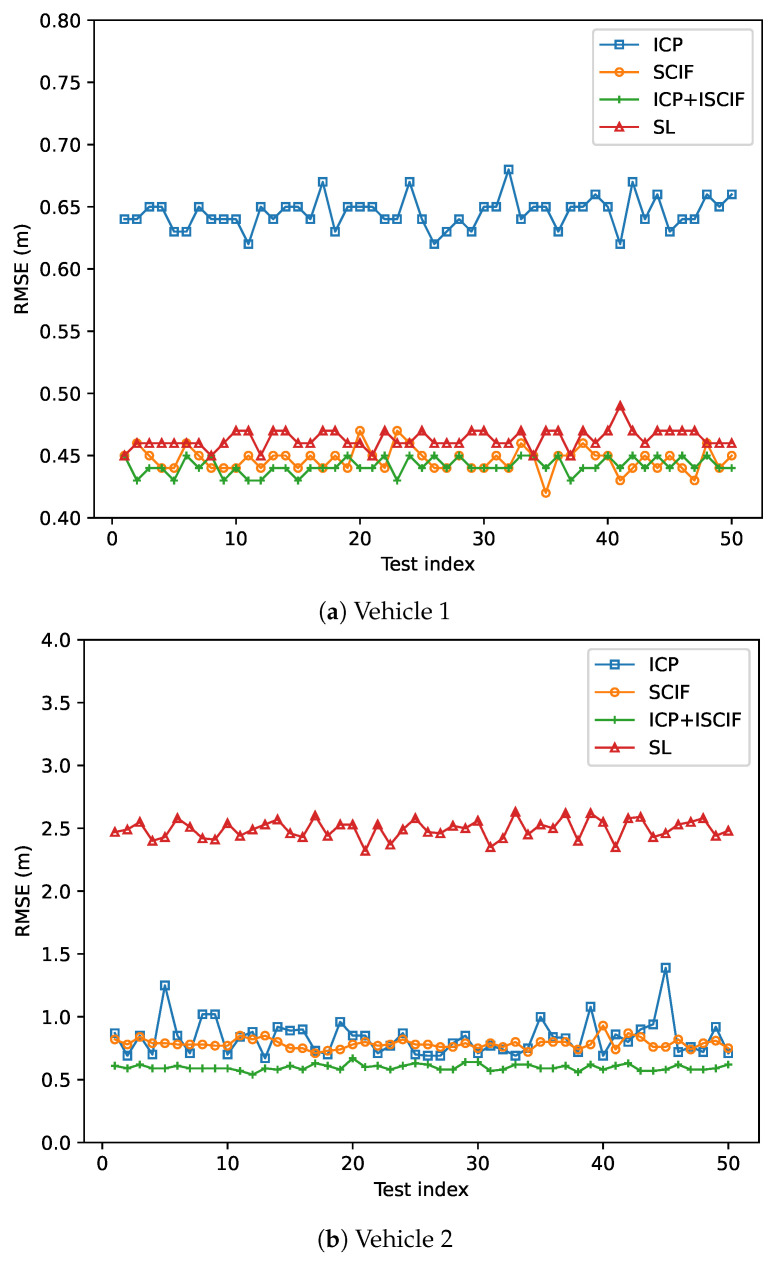

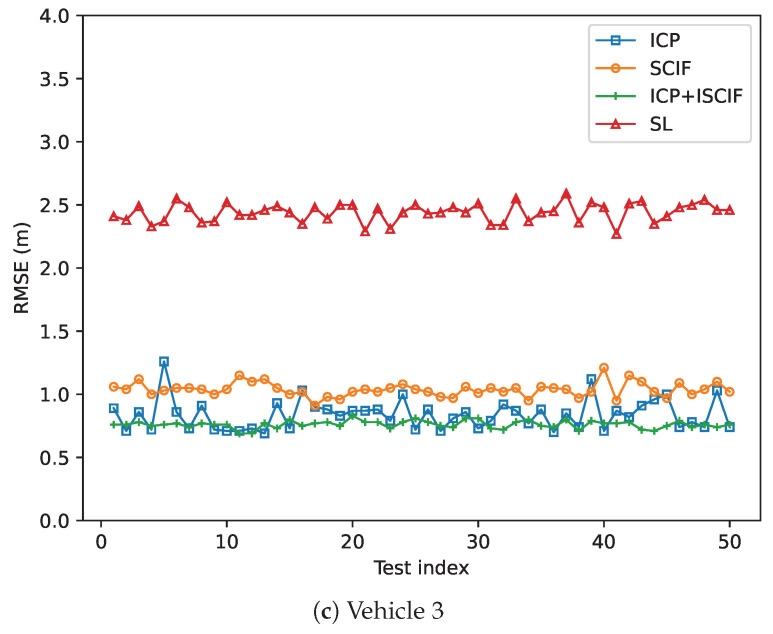
RMSE of vehicles associated with different methods (Vehicle 1 has excellent positioning ability).

In addition, the statistical RMSE results based on 50 experiments are shown in Figure 14. The average RMSE of the ICP method for all vehicles is 0.77 m, which benefits the most compared with the result in previous scenario (shown in Figure 11). However, at certain steps, the true position may not be included in the estimated interval. The average RMSEs of the SL, SCIF, and ICP+ISCIF methods are 1.8 m, 0.76 m, and 0.6 m, respectively. Thus, compared to the SCIF method, our proposed method (ICP+ISCIF) can reduce the RMSE by about 21% in Scenario 2.

### 4.5. Computational Burden Analysis

The matrix operation complexity is shown in Table 2, noting that performing addition, multiplication, and inverse operations with an interval matrix incurs the same level of complexity as with regular matrices. So, by using big *O* notation, the complexity of both ISCIF and SCIF algorithms are $O(n^{3})$ (where *n* is the dimension of the covariance matrix), which depends on the inverse operation of the matrix.

**Figure 15:**
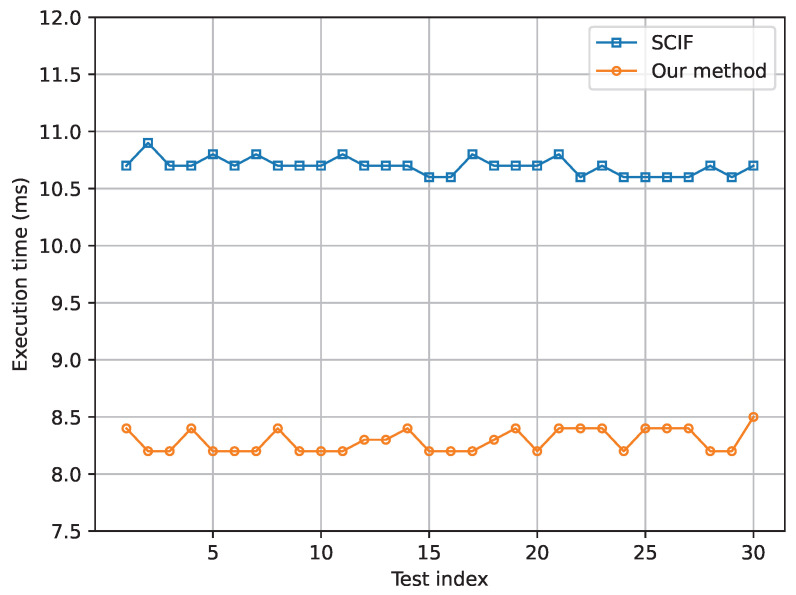
The execution time of SCIF and our method.

Moreover, in order to further investigate the computational overhead of our proposed algorithm, experiments based on execution time are conducted. The result is shown in Figure 15, where the average execution time per time step of SCIF and our method based on 30 experiments are 10.70 ms and 8.29 ms, respectively. Compared to the SCIF method, our method achieves a reduction in execution time of approximately 23%.

### 4.6. Discussion

The statistical results of the two different simulation scenarios have been presented in the previous two subsections. The simulation results show that our proposed method (ICP+ISCIF) can effectively reduce the RMSE of localization results, and the true position can be included in the estimated intervals almost at all times. Moreover, the results of Scenario 2 highlight the significant advantages of cooperative localization systems in terms of accuracy.

Furthermore, we calculate the average rate across all vehicles when the true position is included in the result intervals for both scenarios, $\rho =\frac{F}{R}\approx 97.2\%$, almost all the time, where *F* is the number of times that the true value is included in the output intervals, and *R* is the total number of estimated positions. The reason the true position is not included all the time is due to the effect of noise. Note that this rate can achieve more than about 99% in the second scenario, which indicates our proposed ICP+ISCIF method effectively captures the true position within intervals when the vehicle has good absolute positioning ability.

Based on our simulation experiment results, our proposed algorithm is more suitable for MSMV vehicle localization in future ITS, especially when the utilized sensors (GPS, IMU, and LiDAR) are in good condition. Additionally, our approach exhibits computational advantages as the number of vehicles in the system increases. However, our algorithm will no longer be applicable when sensors are affected by external environmental factors, such as GPS-denied scenarios [28,29].

Furthermore, potential issues may include: our algorithm may become ineffective when GPS is affected by satellite signal strength. One possible solution is to establish sensor fault detection and response mechanisms to promptly adjust vehicle localization accuracy [15]. These adjustments involve employing vehicle localization algorithms based on IMU and LiDAR to mitigate the low accuracy impact caused by GPS sensors [30].

Details of impact on correlated coefficients in Equations (48) and (51) on localization accuracy based on 10 experiments are illustrated in Table 3. Based on the results, we can conclude that different interval width values have a certain impact on localization accuracy, and our algorithm achieves higher accuracy when all interval width values satisfy $\alpha_{xj}=3.5$, $\alpha_{yj}=3.5$, $\alpha_{\theta j}=0$, $\beta_{xi}=0.2$, $\beta_{yi}=0.2$, and $\beta_{\theta i}=0$, respectively.

## 5. Conclusions and Future Work

In order to address the data incest problem in the MSMV localization system, a new data fusion algorithm called ISCIF is proposed, and the generic consistency proof of the ISCIF is provided. Then, the proposed ISCIF is implemented in both absolute and relative positioning stages in a decentralized MSMV localization system. Note that, in the relative positioning stage, since there are multiple relative estimates, which reduce the localization accuracy and increase the computational overhead, the ICP method is employed to mitigate the impact caused by the multiple relative position estimates and provide input data for ISCIF.

Furthermore, the main reasons the ICP method can output accurate results include accurate modeling and constraint propagation. In terms of accurate modeling, the ICP method can precisely infer the possible ranges of variables by considering the relationships between constraints and variables. Additionally, constraint propagation is employed to narrow down the ranges of variables, effectively reducing the solution space and achieving accurate vehicle positioning. Regarding the proposed ISCIF algorithm, it addresses the impact of relative estimation among neighbors by utilizing interval analysis techniques, allowing it to provide state estimation intervals instead of traditional point estimates. This allows the algorithm to more comprehensively represent the uncertainty of state variables and mitigate the impact of state estimation uncertainty. Furthermore, interval arithmetic operations, compared to traditional numerical computations, can reduce rounding errors to some extent, thereby yielding more accurate results.

The proposed method is evaluated through simulation tests with two different scenarios. Based on the results obtained from the simulations, our proposed method can achieve reasonable accuracy. Compared with the SCIF method, our proposed approach can reduce the RMSE by 11% and 21% in Scenario 1 and Scenario 2, respectively. Moreover, the true vehicle position can be included in the intervals output by our method almost all the time, which means that our method can be implemented in the interval non-linear system. Furthermore, compared with the IKF method, our method does not require the inversion of the interval matrix, which avoids potential problems that could affect data fusion accuracy.

Finally, in our future work, we aim to propose an algorithm suitable for GPS-denied scenarios, as GPS signals can easily be affected by satellite transmission signal strength in the real world. Additionally, we intend to test the performance of our proposed method using real-world data.

## Figures and Tables

**Table 1:** Details of parameters.

Parameters	Values
Discrete time step	0.1 (s)
Simulation duration	120 (s)
Velocity of vehicles	15 (m/s)
Velocity standard error	0.2 (m/s)
Direction standard error	0.3 (degrees)
Relative distance standard error	0.2 (m)
Relative orientation standard error	0.1 (degrees)
Absolute positioning standard error on *x*-axis	5 (m)
Absolute positioning standard error on *y*-axis	5 (m)
$\alpha_{xj}$, $\alpha_{yj}$	3.5 (m)
$\beta_{xj}$, $\beta_{yj}$	0.2 (m)
$\alpha_{\theta j}$, $\beta_{\theta i}$	0
TDMA frequency band	5.9 (GHz)
TDMA frame period	100 (ms)

**Table 2:** Matrix operation complexity.

Matrix Operation	Matrix Dimension	Complexity
X+Y	[a∗b]+[a∗b]	$O\left(ab\right)$
X.Y	[a∗b]. [b∗c]	$O\left(abc\right)$
$X^{-1}$	[a∗a]	$O\left(a^{3}\right)$

**Table 3:** The influence of different variables on localization accuracy based on data from 10 experiments.

Para.	RMSEs	R1	R2	R3	R4	R5	R6	R7	R8	R9	R10
$\alpha_{xj,yj}=3$	V1	1.43	1.39	1.36	1.33	1.37	1.34	1.42	1.38	1.33	1.44
$\beta_{xi,yi}=0.1$	V2	1.41	1.41	1.44	1.4	1.43	1.35	1.44	1.49	1.36	1.44
$\alpha_{\theta j},\beta_{\theta i}=0$	V3	1.47	1.48	1.46	1.48	1.48	1.43	1.51	1.54	1.4	1.5
$\alpha_{xj,yj}=3$	V1	1.44	1.32	1.38	1.38	1.39	1.43	1.44	1.42	1.34	1.36
$\beta_{xi,yi}=0.2$	V2	1.51	1.4	1.42	1.46	1.44	1.44	1.46	1.43	1.4	1.42
$\alpha_{\theta j},\beta_{\theta i}=0$	V3	1.54	1.46	1.47	1.52	1.52	1.49	1.51	1.49	1.46	1.47
$\alpha_{xj,yj}=3$	V1	1.41	1.33	1.35	1.33	1.3	1.35	1.3	1.46	1.36	1.35
$\beta_{xi,yi}=0.3$	V2	1.57	1.43	1.42	1.47	1.36	1.46	1.45	1.46	1.54	1.4
$\alpha_{\theta j},\beta_{\theta i}=0$	V3	1.61	1.42	1.41	1.45	1.36	1.44	1.42	1.48	1.5	1.4
$\alpha_{xj,yj}=3.5$	V1	1.41	1.37	1.4	1.38	1.33	1.36	1.37	1.38	1.38	1.32
$\beta_{xi,yi}=0.1$	V2	1.46	1.46	1.41	1.44	1.39	1.43	1.48	1.41	1.37	1.39
$\alpha_{\theta j},\beta_{\theta i}=0$	V3	1.54	1.53	1.45	1.49	1.43	1.45	1.49	1.48	1.46	1.48
$\alpha_{xj,yj}=3.5$	V1	1.37	1.36	1.36	1.31	1.36	1.38	1.36	1.3	1.36	1.39
$\beta_{xi,yi}=0.2$	V2	1.38	1.45	1.37	1.36	1.48	1.42	1.43	1.34	1.38	1.39
$\alpha_{\theta j},\beta_{\theta i}=0$	V3	1.46	1.5	1.42	1.42	1.54	1.48	1.5	1.39	1.46	1.44
$\alpha_{xj,yj}=3.5$	V1	1.45	1.33	1.38	1.53	1.43	1.47	1.32	1.36	1.34	1.6
$\beta_{xi,yi}=0.3$	V2	1.52	1.41	1.43	1.55	1.42	1.49	1.39	1.46	1.47	1.52
$\alpha_{\theta j},\beta_{\theta i}=0$	V3	1.57	1.47	1.49	1.58	1.45	1.54	1.44	1.52	1.53	1.59
$\alpha_{xj,yj}=4$	V1	1.4	1.35	1.36	1.39	1.46	1.35	1.33	1.45	1.4	1.43
$\beta_{xi,yi}=0.1$	V2	1.41	1.49	1.49	1.35	1.51	1.37	1.41	1.49	1.49	1.45
$\alpha_{\theta j},\beta_{\theta i}=0$	V3	1.46	1.57	1.55	1.44	1.57	1.44	1.47	1.53	1.54	1.46
$\alpha_{xj,yj}=4$	V1	1.34	1.41	1.38	1.35	1.38	1.4	1.51	1.41	1.45	1.35
$\beta_{xi,yi}=0.2$	V2	1.41	1.48	1.41	1.59	1.46	1.38	1.47	1.48	1.47	1.39
$\alpha_{\theta j},\beta_{\theta i}=0$	V3	1.48	1.53	1.44	1.65	1.48	1.42	1.53	1.5	1.53	1.45
$\alpha_{xj,yj}=4$	V1	1.48	1.38	1.41	1.45	1.39	1.36	1.39	1.31	1.42	1.35
$\beta_{xi,yi}=0.3$	V2	1.49	1.46	1.48	1.45	1.44	1.41	1.45	1.4	1.5	1.47
$\alpha_{\theta j},\beta_{\theta i}=0$	V3	1.54	1.52	1.54	1.5	1.51	1.47	1.5	1.48	1.56	1.51

## Data Availability

Some or all data, models, or code that support the findings of this study are available from the corresponding author upon reasonable request.
